# Quantitative sensorische Testung im Rahmen neuropathischer Schmerzen und ihre Bedeutung für die Physiotherapie

**DOI:** 10.1007/s00482-021-00576-z

**Published:** 2021-08-23

**Authors:** Magdalena Adler, Bernhard Taxer

**Affiliations:** 1 Graz, Österreich; 2 Fachhochschule für angewandte Wissenschaft, FH JOANNEUM Graz, Eggenberger Allee 13, 8010 Graz, Österreich

**Keywords:** Physiotherapie, Polyneuropathie, Schmerz, Kälte- und Wärmedetektionsschwellen, Bed-side Untersuchung, Physiotherapy, Polyneuropathy, Pain, Cold- and heat detection threshold, Bed-side assessment

## Abstract

**Hintergrund:**

Neuropathische Schmerzsyndrome zeichnen sich durch hohe Chronifizierungsraten sowie lange und intensive Schmerzepisoden aus. Ein treffsicheres Erkennen stellt eine Grundkompetenz von Physiotherapeuten dar, ermöglicht eine ursachengerechte Therapie und kann die Entstehung von Folgeschäden verhindern. Die quantitative sensorische Testung (QST) wird im medizinischen Rahmen als Ergänzung zur klinischen Sensibilitätsprüfung eingesetzt, konnte inzwischen eine beachtliche Stellung in der Forschung einnehmen, wird in der klinischen Praxis jedoch weniger häufig eingesetzt.

**Fragestellung:**

Welchen Mehrwert hat die QST in der Untersuchung neuropathischer Schmerzen? Was sind die Ursachen für die begrenzte klinische Anwendung der QST? Was sind potenzielle Wege für einen erfolgreichen Übertrag der QST in die physiotherapeutische Praxis?

**Methode:**

Literaturrecherche im Zuge einer Bachelorarbeit Physiotherapie.

**Ergebnisse:**

Als valides Untersuchungsinstrument, das zur Evaluierung des gesamten somatosensorischen Profils geeignet ist, bietet die QST vor allem im Bereich der Small-fibre-Neuropathien einen erheblichen Vorteil gegenüber konventionellen Testverfahren. Diese kleinen Fasern scheinen insbesondere in der Frühphase von Neuropathien betroffen zu sein und können über konventionelle Testverfahren nicht evaluiert werden. Das macht den Einsatz von Teilaspekten der QST zu einem nützlichen Instrument für Physiotherapeuten und medizinisches Personal, was besonders in der Früherkennung von Neuropathien von großem Nutzen ist.

**Diskussion:**

Trotz des bestehenden großen Nutzens existieren bis dato noch Limitationen, die den klinischen Routineeinsatz der QST behindern. Einige davon können durch exakte Testausführungen und Vorkehrungen bis zu einem gewissen Grad überwunden werden, andere, für die Klinik hochrelevante Bereiche wie die hohen Anschaffungskosten der Geräte und der hohe zeitliche Aufwand der Durchführung konnten bislang noch nicht zufriedenstellend gelöst werden. Weniger umfassende Testprotokolle sowie die Entwicklung handlicher und kostengünstiger Testgeräte könnten diesbezüglich erste Lösungsansätze darstellen. Die Ergänzung der konventionellen Bedside-Untersuchung um Testungen zur Wärmesensibilität und Schmerzschwellenbestimmung kann eine weitere Möglichkeit darstellen, um den dargestellten Mehrwert der QST in den klinischen Alltag zu integrieren.

**Schlussfolgerung:**

Die QST steuert einen wesentlichen Beitrag zur Untersuchung und Diagnose von Neuropathien bei. Physiotherapeuten sind dazu angehalten, Teilaspekte aus der QST in eine standardmäßige Untersuchung zu implementieren, um sowohl in der Früherkennung als auch in der Behandlung positiv einzuwirken.

## Abstract (en)

**Background:**

Neuropathic pain syndromes are typically characterized by high chronification rates as well as long and intensive pain episodes. Early and accurate diagnosis of neuropathic pain is a basic skill of physiotherapists and other medical professionals, may allow for appropriate medical treatment and help to prevent possible consequential damage. Quantitative sensory testing (QST) can be applied as a supplement to conventional neurological bedside testing in the evaluation of neuropathic pain. Over recent decades, QST has come to hold a significant position in the field of pain research. However, despite these developments, the application of QST in clinical practice has lagged behind.

**Objectives:**

What is the value of QST in the study of neuropathic pain? Have the conditions for personal clinical use of the QST been established in physiotherapy practice? Have the pathways for specific implementation of the QST been defined?

**Method:**

Literature research as part of a Bachelor thesis in Physiotherapy.

**Results:**

QST constitutes a valid examination tool that is able to evaluate the complete somatosensory profile. In this way, QST may provide substantial additional benefit in the examination of neuropathic pain patients compared to other conventional testing procedures, especially when it comes to small-fibre neuropathies. These small fibres seem to be particularly affected in asymptomatic patients as well as early phases of neuropathies and cannot be investigated via conventional testing procedures. This makes the use of partial aspects of the QST a proven instrument for physiotherapists and medical staff, which was particularly useful in the decision-making process for neuropathies.

**Discussion:**

Nonetheless, regarding the results, there are still several limiting factors that hamper the routine use of QST. Some of these can be resolved by precisely adhering to testing protocols and taking precautions. Other highly relevant issues for clinical practice, such as the immense cost of equipment and the excessive time required for testing, have not been satisfactorily overcome as yet. Less comprehensive testing protocols as well as the innovation of handy and cost-effective testing devices might offer initial approaches to enhance the widespread use of QST. Complementing conventional bedside testing by adding thermal discrimination tests and pain detection threshold tests might prove to be another possibility to integrate the benefit of QST into clinical practice.

**Conclusion:**

QST makes a significant contribution to the investigation and diagnosis of neuropathies. Physiotherapists are encouraged to implement partial aspects of the QST in a standard examination in order to have a positive effect on both early detection and treatment.

## Einleitung

Die Prävalenz neuropathischer Schmerzen in der allgemeinen Bevölkerung liegt laut Studien zwischen 6,9 und 10 % [31]. Neuropathischer Schmerz zeichnet sich durch eine rasche Chronifizierung aus [5, 15, 32]. Der enge Zusammenhang zwischen chronischen und neuropathischen Schmerzen wird durch epidemiologische Umfragestudien belegt, welche davon ausgehen, dass etwa 20 % der chronischen Schmerzpatienten neuropathische Schmerzcharakteristika aufweisen [10, 63]. Zusätzlich zeigen sich signifikant höhere Schmerzintensitäten und längere Schmerzepisoden. Unabhängig von dem direkt durch die Schmerzen verursachten Leiden kommt es häufiger zu Komorbiditäten wie Depressionen oder Schlafstörungen [19, 52]. Zusammenfassend wirkt sich der neuropathische Schmerz für das Individuum oft in einem hohen Maß an Einschränkungen sowie einer deutlichen Reduktion der Lebensqualität aus [27, 45, 63]. Physiotherapeuten befassen sich sowohl aus klinischer als auch aus wissenschaftlicher Sicht mit diesem komplexen klinischen Bild [34, 44, 59]. Dabei zeigt sich, dass einerseits die Untersuchung und Testung und andererseits die Behandlung und das Management nicht nur die Betroffenen vor Herausforderungen stellt, sondern auch die jeweiligen Behandler.

## Klinische Relevanz

Faktoren wie die oben angeführten unterstreichen die Wichtigkeit einer akkuraten und frühzeitigen Identifizierung von neuropathischen Schmerzen. Eine treffsichere Diagnostik bildet die Grundlage für eine möglichst zielgerichtete und ursachengerechte Therapie. Der neurologischen Untersuchung und insbesondere der Sensibilitätsprüfung wird bei der Untersuchung und Diagnostik neuropathischer Schmerzen eine zentrale Bedeutung beigemessen. Diese wird in der klinischen Praxis meist mit relativ einfachen Untersuchungsutensilien durchgeführt und als neurologische *Bedside-Untersuchung* (BU) bezeichnet. Die Hauptlimitationen dieser traditionellen Untersuchungen liegen in der zumeist qualitativen Erfassung von Parametern und dem Fehlen von standardisierten Kontrollvariablen und Reizintensitäten. In diesem Zusammenhang stellt die quantitative sensorische Testung (QST) ein Verfahren dar, das diese Limitationen kompensieren kann. Es handelt sich dabei um eine standardisierte klinische Sensibilitätsprüfung, bei der verschiedene Reize unter einem vorgegeben Testprotokoll verabreicht werden. Auf diese Weise werden die individuellen Wahrnehmungs- und Schmerzschwellen der Testpersonen erhoben. Durch den Vergleich der Messwerte mit vorgegebenen Normwerten kann Auskunft über das Vorhandensein von Plus- oder Minusphänomenen gewonnen werden [43]. Die QST wird als Ergänzung zur traditionellen neurologischen BU empfohlen und deren Vorteile werden vor allem in der präziseren Erfassung der somatosensorischen Funktionen gesehen [30, 65].

Die QST konnte im Rahmen der Forschung enorm an Bedeutung gewinnen, ihre Implementierung in der klinischen Praxis ist jedoch noch nicht gleichermaßen vorangeschritten [7]. Auch wenn die QST in ihrem Ablauf anderen im klinischen Alltag sehr gängigen Testverfahren wie der Gesichtsfeld- oder Sehstärkemessung gleicht, konnte sie im klinischen Setting noch nicht recht Fuß fassen und muss sich hinsichtlich ihrer klinischen Relevanz hinter konventionellen neurophysiologischen Testverfahren wie der Nervenleitgeschwindigkeitsmessung (NLG) oder der intraepidermalen Nervenfaserdichtemessung (IENFD) reihen [3].

Diese Arbeit versucht den potenziellen Mehrwert der QST vor allem in der physiotherapeutischen Untersuchung von neuropathischen Schmerzpatienten darzulegen und diesen den eventuellen Schwierigkeiten, die beim Einsatz von QST auftreten könnten, gegenüberzustellen. Des Weiteren wird versucht, die Ursachen für die begrenzte klinische Anwendung zu identifizieren und vor allem die potenzielle klinische Relevanz für den physiotherapeutischen Alltag aufzuzeigen.

## Neuropathischer Schmerz und Physiotherapie

Neuropathische Schmerzsyndrome treten nach einer Erkrankung oder Läsion des somatosensorischen Nervensystems auf [21]. Im Unterschied zum nozizeptiven Schmerz, bei dem hochschwellige Reize über prinzipiell intakte somatosensorische Fasern weitergleitet werden, liegt die primäre Ursache der neuropathischen Schmerzen in einer Läsion oder Erkrankung des somatosensorischen Systems selbst. Ektopische Aktivitäten, periphere und zentrale Sensibilisierung, defizitäre inhibitorische Modulation sowie die Aktivierung von Mikroglia scheinen einige der grundlegenden Wirkmechanismen bei der Entstehung neuropathischer Schmerzen zu sein [15, 25]. Diese Mechanismen sind sehr komplex und können äußerst variabel in Erscheinung treten.

Unter dem Überbegriff „neuropathische Schmerzen“ kann eine breite Palette an klinischen Zuständen subsumiert werden, welche nach anatomischen (peripher oder zentral) und ätiologischen Gesichtspunkten eingeteilt werden können. Tab. 1 bildet eine solche Kategorisierung ab, wobei zusätzlich charakteristische Beispiele an Grunderkrankungen angeführt werden.

**Table 1:** 

Ursache	Peripher	Spinal	Gehirn
Genetisch	Fabry-Neuropathie	Syringomyelie	Syringobulbie
Metabolisch	Schmerzhafte DPN	B12-Myelopathie	–
Traumatisch	Nervenläsion	Rückenmarksverletzung	Schädel-Hirn-Trauma (SHT)
Vaskulär	Vaskulitische Neuropathie	Rückenmarksinsult	Insult
Neoplastisch	Tumorkompressionsneuropathie, z. B. gutartiges Lipom im Karpaltunnel	Tumorkompression, z. B. Meningeom	Tumorkompression, z. B. Glioblastom
Immunologisch	Guillain-Barré-Syndrom	Multiple Sklerose	Multiple Sklerose
Infektiös	HIV, Borreliose	Infektiöse Myelitis	Enzephalitis
Toxisch	Chemotherapeutisch oder alkoholinduzierte Neuropathie	–	–

*DPN* diabetische Polyneuropathie

Zu den in der Physiotherapie häufigsten und relevantesten neuropathischen Schmerzsyndromen zählen [15]:

- Zervikale und lumbale Radikulopathie bzw. radikuläre Schmerzen,
- Schmerzen nach Verletzungen peripherer Nervenstrukturen,
- Diabetische Polyneuropathie (DPN),
- Phantomschmerzen,
- Zentrale neuropathische Schmerzen im Zuge eines Schlaganfalls, multipler Sklerose oder einer Verletzung des Rückenmarks oder Gehirns.

Diese Darstellung allein zeigt noch nicht die klinische Relevanz einer physiotherapeutischen Rehabilitation. Die in der Grundlagenforschung bis dato beschriebenen Pathomechanismen, welche sowohl periphere als auch zentrale neuroimmunologische Mechanismen inkludieren [2, 54, 56, 60, 67], liefern jedoch Hinweise, dass bewegungs- und manualtherapeutische Ansätze tendenziell einen positiven Beitrag aus dem nichtpharmakologischen Behandlungsspektrum liefern können [16, 20, 34, 36, 53]. Nicht zu unterschätzen ist im Rahmen einer physiotherapeutischen Anamnese und Untersuchung die Früherkennung im Sinne eines sekundärpräventiven Ansatzes, wie dies zum Beispiel bei Patienten, welche an Diabetes mellitus erkranken, der Fall sein kann.

## Physiotherapeutische Untersuchung und Diagnostik neuropathischer Schmerzen

Ähnlich dem medizinischen Vorgehen in Bezug auf die Diagnose von neuropathischen Schmerzen stellen auch für die Physiotherapie die Abgrenzung zu anderen Schmerzmechanismen sowie der Nachweis der Läsionen, die für den Schmerz verantwortlich sind, große Herausforderungen dar [9], sind aber für das weitere therapeutische Management unumgänglich. Dabei messen derzeit gültige Leitlinien einer gezielten Anamnese inklusive detaillierter Schmerzerhebung wesentliche Bedeutung bei. Liegt das Leitsymptom Schmerz vor und lässt sich dieser in einem neuroanatomisch plausiblen Gebiet lokalisieren, weisen die erhobenen Informationen auf die Arbeitshypothese „neuropathischer Schmerz“ hin. In der klinischen Untersuchung wird diese Hypothese mithilfe von verschiedenen Untersuchungsschritten bestätigt oder verworfen. Dabei bildet die Sensibilitätsprüfung eine zentrale Säule, welche sensorische Funktionsdefizite und -zuwächse, die sogenannten Plus- oder Minusphänomene, erfassen soll [5, 9, 25, 27, 65].

In der klinischen Praxis kann diese Sensibilitätsprüfung gemeinsam mit der Anamnese bereits ausreichend sein, um neuropathisches Schmerzgeschehen feststellen zu können bzw. um von „wahrscheinlich vorhandenem“ neuropathischem Schmerz sprechen zu können. Um diesen Verdacht zu erhärten, bedarf es jedoch des Nachweises der Läsion oder Erkrankung, auf die die Schmerzen zurückzuführen sind. Dies kann häufig nur mithilfe von apparativen Verfahren wie der IENFD, somatosensibel evozierten Potenzialen (SEP), NLG oder Magnetresonanzbildern (MRT) gelingen, welche in der Regel ärztlichen Kompetenzen vorbehalten sind [27, 65].

**Figure 1:**
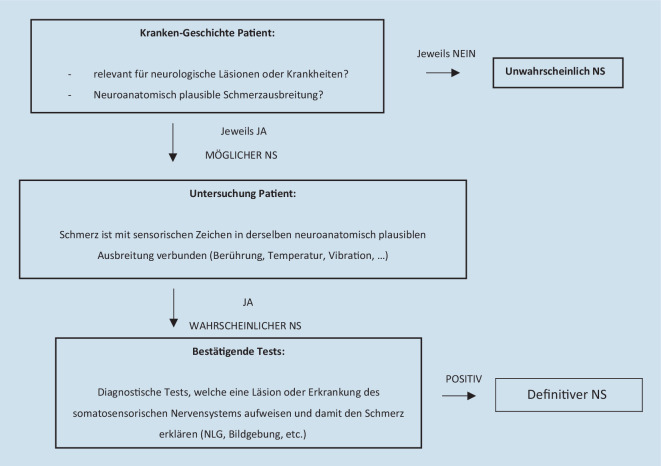

Abb. 1 zeigt einen möglichen Ablauf der Untersuchungsschritte bei neuropathischen Schmerzen, wie sie von aktuellen Leitlinien empfohlen werden [21].

Physiotherapeuten sind primär auf eine klinische Untersuchung inklusive umfassender Schmerzanamnese angewiesen, um eine Arbeitshypothese zu erfassen. Es ergibt sich nun die Überlegung, welchen Mehrwert Therapeuten aus Erkenntnissen zur QST ziehen können und in weiterer Folge als Parameter in die klinische Untersuchung standardisiert integrieren könnten.

## Mehrwert der quantitativen sensorischen Testung

Die QST stellt eine standardisierte klinische Sensibilitätsprüfung dar, die die somatosensorische Funktion einer vordefinierten Köperstelle quantitativ beurteilt. Dabei werden thermische, elektrische und mechanische Reize in auf- oder absteigender Intensität auf die Haut aufgetragen. Diese sind in ihrer Modalität, Intensität und zeitlichen sowie räumlichen Eigenschaft exakt kalibriert und können gezielt bestimmte Nervenfasern und -bahnen ansprechen. Über die Rückmeldung der Probanden werden die individuellen Wahrnehmungs- und Schmerzschwellen für die jeweiligen Reize bestimmt. Als psychophysisches Testverfahren stellt die QST ein semiobjektives Verfahren dar, da die Schwellenwerte auf Basis der subjektiven Einschätzungen der Wahrnehmungs- und Schmerzzeitpunkte der Testpersonen festgelegt werden [18, 23, 57].

Durch den Vergleich der erhobenen Schwellenwerte mit vordefinierten Normwerten kann Auskunft über die Funktionalität des somatosensorischen Systems gewonnen werden [3, 43, 57]. Hyperalgesien und mechanische Allodynien stellen dabei sogenannte Gain-of-function-Phänomene dar [42, 55], welche sich in herabgesetzten QST-Schmerzschwellen darstellen würden. Im Vergleich zu anderen apparativen Testverfahren zur Untersuchung neuropathischer Schmerzen bietet die QST als einzige die Möglichkeit, unter anderem eben diese Phänomene auf quantitativer Ebene zu erheben. Mit dieser spezifischen Fähigkeit konnte ein wesentlicher Beitrag zur laboratorischen Schmerzforschung der letzten Jahrzehnte geleistet werden. Trotzdem stößt die routinemäßige Anwendung dieses Tools in der Praxis bislang noch nicht auf breite Akzeptanz. Außerdem ist die genaue Lokalisation des Ursprungs der Nervenschädigung mittels QST nicht möglich [3, 7, 43].

**Figure 2:**
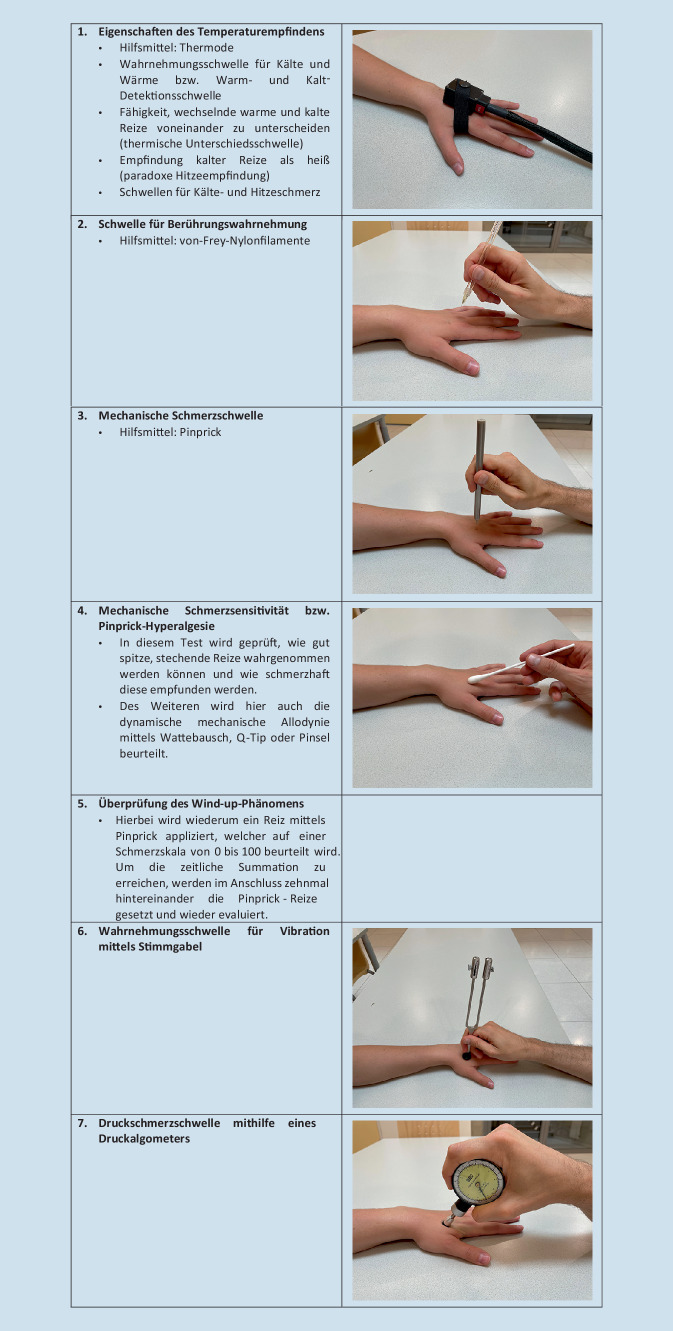

Eine technische Herausforderung der QST stellt das Festlegen von akkuraten und reproduzierbaren Schwellenwerten dar, die in einem vernünftigen Zeitumfang erhoben werden können. Zur Erleichterung dafür wurden Testalgorithmen eingeführt [57]. Ein relativ kurzes und umfassendes Testprotokoll wurde 2006 vom Deutschen Forschungsverband Neuropathischer Schmerz (DFNS) entwickelt. Dieses Protokoll beinhaltet die Überprüfung des vollständigen somatosensorischen Profils, das in sieben Testungen insgesamt dreizehn verschiedene Parameter erfasst. Durch die Erhebung von thermischen und mechanischen Schmerz- und Wahrnehmungsschwellen können sowohl die kleineren als auch die größeren Fasern des somatosensorischen Systems evaluiert werden. Die Dauer der gesamten Testbatterie beträgt für eine Körperstelle etwa 30 min und ungefähr eine Stunde, wenn zusätzlich eine symptomfreie Stelle zur Kontrolle untersucht wird [51]. Abb. 2 soll diesen Testablauf zum besseren Verständnis darstellen.

Die QST wird von derzeitigen „guidelines“ als möglicher Untersuchungsschritt bei der Beurteilung neuropathischer Schmerzen vorgeschlagen. In diesen Empfehlungen wird stets die Wichtigkeit der neurologischen BU betont, welche einer QST vorangehen sollte und als wesentlicher Bestandteil der Diagnostik von neuropathischen Schmerzen betrachtet wird [27]. Im Zuge der BU werden jene Bereiche mit sensorischen Abweichungen erfasst, welche im nachfolgenden Schritt als Untersuchungsstellen der QST dienen. Die begrenzte Verfügbarkeit von Faktoren wie Zeit oder Aufmerksamkeitsspanne der Patienten erfordert eine Eingrenzung der Teststellen [4].

## Diskussion

Mit weltweit circa 425 Mio. betroffenen Diabetikern [33] stellt diese Erkrankung eine der häufigsten Gründe für die Entwicklung von Neuropathien dar [11]. Bis zu mehr als 50 % der Betroffenen entwickeln neuropathische Schmerzsymptome [1, 29]. Dementsprechend umfassend ist daher auch die QST im Rahmen schmerzhafter Neuropathien bei DM untersucht worden.

Im Zuge der Untersuchungen von symptomatischen versus asymptomatischen diabetischen Neuropathien zeigt sich, dass insbesondere die thermische Komponente der QST bei Personen mit asymptomatischem oder minimal symptomatischem DM häufiger Abnormitäten aufweist als die vibratorische Reizsetzung oder die NLG. Dies betrifft auch asymptomatische Patienten, bei denen die NLG noch gar keine Normabweichungen entdecken konnte [12]. Weintrob et al. (2007) zeigen, dass die QST signifikante Unterschiede zur untersuchten Kontrollgruppe in den Kategorien Vibration, Wärme und Kälte aufweist. Interessanterweise klagten dabei nur 11 % der untersuchten Diabetiker zum Zeitpunkt der Untersuchung über neuropathische Symptome [66].

IENFD und QST sind in der Lage, Neuropathien bei Diabetikern mit normaler NLG zu erkennen. Bezüglich asymptomatischer Diabetiker konnten sowohl die IENFD als auch die Kälteschwellenbestimmung der QST signifikante Unterschiede zur gesunden Kontrollgruppe feststellen, während die Wärmeschwellenmessung diesbezüglich ergebnislos ausfiel [41]. Diese Unterschiede in Bezug auf abnormale sensorische Profile, bei gleichzeitig unauffälliger NLG, wurden an anderer Stelle bestätigt. Dabei wurden bei 98 % der Diabetiker mit normaler NLG signifikant erhöhte Wärmedetektionsschwellen gemessen [35].

Bei schmerzhaften versus nichtschmerzhaften diabetischen Neuropathien konnte die QST, mit Ausnahme einer untersuchten Studie, signifikant höhere Vibrations- und vor allem Temperaturdetektionsschwellen bei Schmerzpatienten feststellen [14]. Kramer et al. (2004) hingegen weisen im Zuge ihrer Untersuchungen keine Unterschiede in diesen beiden Aspekten auf. Es zeigte sich jedoch eine negative Korrelation zwischen der Schmerzintensität und der Kältedetektionsschwelle. Dieselbe negative Korrelation ließ sich auch für die Wärmedetektionsschwelle und die Schmerzintensität nachweisen [37].

Dass vor allem die Untersuchung von Wärme- bzw. Kälteschmerzschwellen eine wesentliche Rolle im Rahmen eines neuropathischen Geschehens spielt, wird in mehreren Studien bestätigt. Dieser Parameter scheint der Schlüsselaspekt im Rahmen sensorischer Dysfunktionen, vor allem bei neuropathischen Geschehen bei Diabetes mellitus zu sein. Auch der Vibrationsuntersuchung wird eine wichtige, wenn auch nicht derartig starke Rolle zugeschrieben. Nichtsdestotrotz zeigt sich eine Überlegenheit im Vergleich zur NLG [12, 35, 37, 66].

Aufgrund der dargestellten Ergebnisse [12, 14, 37, 41] wird die QST von der Peripheral Neuropathy Association und der American Diabetes Association als geeignete Untersuchung von Patienten mit DPN empfohlen. Insgesamt konnte die QST in den meisten Studien bei fortgeschrittenem Erkrankungsstadium bzw. längerer Erkrankungsdauer höhere Signifikanzen und diagnostische Sensitivität erzielen [12, 14, 35, 41]. Es lässt sich erschließen, dass die thermische QST vor allem in den Anfangsstadien von Neuropathien ein effektives Messverfahren darstellt und dass in diesen frühen Stadien vorwiegend kleinere, unmyelinisierte Fasern betroffen zu sein scheinen. Die Vermutung, dass eine Dysfunktion der kleinen Fasern ein frühes Anzeichen für eine (subklinische) DPN sein kann, wurde bereits von anderen Autoren aufgestellt und bestätigt [6, 39].

Mit dem Hintergrund der Annahme, dass hauptsächlich kleinere Fasern in der Frühphase von Neuropathien betroffen sind, scheint die thermische QST somit besonders gut dazu geeignet zu sein, asymptomatische bzw. subklinische DPN zu erkennen. Insgesamt scheinen die unterschiedlichen Erkrankungsstadien ein Grund für die divergierenden Ergebnisse zu sein [14].

Es ist jedoch festzuhalten, dass ein fortgeschrittenes Erkrankungsstadium nicht immer gleichbedeutend mit stärker ausgeprägten Symptomen sein muss [66]. Außerdem deuten einige Ergebnisse darauf hin, dass das Vorhandensein einer Schmerzsymptomatik bei neuropathischen Krankheitsbildern mit einer Funktionsstörung kleinerer Fasern einhergeht [37]. Aus diesen vielfältigen Erscheinungsbildern neuropathischer Schmerzsyndrome geht hervor, dass eine periphere Nervenschädigung allein noch nicht zur Erklärung von neuropathischen Schmerzen ausreicht. Insgesamt ist daraus abzuleiten, dass die DPN eine sehr heterogene Erkrankung darstellt, bei der verschiedene Fasern in unterschiedlichem Ausmaß betroffen sein können und deren Progression nicht nur vom zeitlichen Umfang bzw. der Dauer der Erkrankung, sondern auch von anderen, zufällig verteilten Variablen abhängig ist [14].

Diese Aspekte lassen den Schluss zu, dass ein abweichendes Funktionsprofil der kleineren Fasern, welche über Temperaturstimuli gereizt werden, eine Art frühes Anzeichen für die Entwicklung neuropathischer Schmerzen sein kann, welches wiederum dafür genutzt werden könnte, Risikopatienten für die Entwicklung neuropathischer Schmerzen zu identifizieren. Dazu können vor allem Physiotherapeuten einen wesentlichen Beitrag leisten. In deren Setting besteht die Möglichkeit, derartige Aspekte zu untersuchen und zu adressieren. Eine dementsprechende Kommunikation mit dem behandelnden Arzt und eine rechtzeitig eingeleitete medikamentöse und nichtmedikamentöse Intervention könnten dabei eine Chronifizierung und Zunahme der Schmerzen bzw. Probleme verhindern.

Zusammenfassend lässt sich ableiten, dass die QST ein geeignetes Messinstrument zur Evaluierung von Patienten mit DPN darstellt, das einen gewissen Vorteil gegenüber anderen Messmethoden bringt. Insbesondere die thermische QST-Messung kann in der Frühphase von Neuropathien nützliche Informationen für Physiotherapeuten und andere medizinische Berufsgruppen bereitstellen und eine wichtige Rolle in der Früherkennung von DPN spielen. Was sind nun die Gründe für die begrenzte klinische Implementierung und wie könnte man diesen entgegenwirken?

### Unzureichende Harmonisierung der Standards

Hierbei spielt vor allem eine klare, schriftliche Standardisierung sowohl der durchgeführten Testung als auch der Instruktion eine Rolle. Es wird mehrfach darauf hingewiesen, eine gute und genaue Dokumentation zu führen, um eine Vergleichbarkeit zuzulassen. Um mögliche Fehlerquellen zu minimieren, empfiehlt es sich, das anwendende Personal zu schulen und die Testumgebung gut und klar zu strukturieren, um mögliche Ablenkungsquellen zu reduzieren. Zertifikatsvergaben für die jeweiligen Labore, Beachten von Standardwerten und eine klare Dokumentation aller möglichen Fehlerquellen gelten als unumgänglich, um diese minimal zu halten [3, 22, 28, 48]. Dass diese Aspekte im Rahmen wissenschaftlicher Untersuchungen per se schon mithilfe von Laborbüchern und Ähnlichem eingehalten werden müssen, entspricht den allgemeinen wissenschaftlichen Kriterien. Für die klinische standardmäßige Einführung im Rahmen des Patientenmanagements stellt sich die Frage, ob es dem Aufwand gerecht wird.

### Fehlender Konsens bei der Interpretation der Ergebnisse

Die in der Praxis herrschende Unsicherheit darüber, wie die in der QST gewonnenen Daten zu interpretieren seien, scheint ein weiterer Faktor zu sein, der die routinemäßige Anwendung in der klinischen Praxis noch behindert. Diese Unsicherheit wird vor allem auf die geringe Anzahl an verfügbaren alters- und geschlechtskorrigierten Normwerten zurückgeführt [4, 47, 51]. Bereits mehrfach haben Studien bestätigt, dass die sensorischen Normwerte einer Person signifikant vom Alter, dem Geschlecht und der Teststelle abhängen [13, 24, 50, 62, 68]. Bereits veröffentlichte Referenzwerte müssen diesen Umstand berücksichtigen, wenn gewährleistet werden soll, dass valide Aussagen aus der QST getroffen werden können [8, 48].

### Unsicherheit über Mehrwert der QST im Vergleich zur Bedside-Untersuchung

In der klinischen Praxis scheint oft noch Unsicherheit darüber zu herrschen, welchen Nutzen die QST im Vergleich zur konventionellen neurologischen BU bieten kann. Wie bereits erwähnt, wird die QST immer nur als Folgeuntersuchung auf eine neurologische Sensibilitätsuntersuchung empfohlen. Während die traditionelle neurologische Untersuchung dazu in der Lage ist, große Symptombereiche qualitativ abzudecken, kann bei der QST aufgrund des erheblichen Zeitaufwands der Testung immer nur eine relativ kleine Körperstelle überprüft werden. Die Auswahl der zu testenden Körperstelle wird über die zuvor erfolgte BU festgelegt und üblicherweise an der Stelle der am stärksten ausgeprägten sensorischen Defizite oder des maximalen Schmerzes durchgeführt [3]. Diese Auswahl der Teststelle stellt einen essenziellen Schritt dar, insbesondere wenn man berücksichtigt, dass sich die Ergebnisse der QST signifikant verändern können, wenn die Teststelle nur um ein kleines Stück verlagert wird [7]. Aus diesem Umstand geht die Wichtigkeit der traditionellen Sensibilitätsuntersuchung hervor und es wird deutlich, dass die QST derzeit nicht als eigenständiges Testverfahren, sondern nur als ergänzendes Untersuchungswerkzeug eingesetzt werden kann [3].

Bislang existieren noch wenige Studien, die das Resultat der neurologischen BU mit jenen der QST direkt vergleichen. Eine Untersuchung aus dem Jahr 2008 legt jedoch nahe, dass die Ergebnisse der beiden Testverfahren nicht immer übereinstimmen müssen. Dabei stellte sich heraus, dass die QST und die BU bei der mechanischen Sensibilitätsprüfung in nur 48 % der Fälle zum selben Ergebnis führten. Die Überprüfung der Kälte- und Wärmesensibilität verhielt sich ähnlich und es wurden bei jeweils 54 % bzw. 58 % der Patienten übereinstimmende Ergebnisse erhoben [40]. Da diese Untersuchung jedoch eine relativ geringe Anzahl an Testpersonen inkludierte und bislang noch keine vergleichenden Studien vorliegen, bedarf es weiterer Forschung in diesem Bereich, um diese Aussagen verallgemeinern zu können. Bis dahin kann die QST als nützliches ergänzendes Testverfahren in der Untersuchung von neuropathischen Schmerzen betrachtet werden, dessen Mehrwert gegenüber der traditionellen Sensibilitätsprüfung vor allem in der präziseren und quantitativen Erfassung des somatosensorischen Profils liegt [30].

### Abhängigkeit der Testergebnisse von psychosozialen Faktoren

Die QST weist aufgrund der Natur ihres Testverfahrens eine erhöhte Anfälligkeit für Verzerrungen durch psychosoziale Faktoren auf, was sowohl eine Herausforderung für die Durchführung als auch für die Interpretation der Testung darstellt. Zwar bedarf es einer möglichst effektiven Adressierung dieser Risikofaktoren, die durch entsprechende Vorsichtsmaßnahmen und Schulung der Beteiligten so weit wie möglich minimiert werden sollten, das alleinige Vorliegen von Risikofaktoren entkräftet jedoch noch nicht den Wert des Verfahrens an sich. Die Entwicklung von Screeningtools, bezogen auf In- und Exklusionskriterien, könnte dabei vor allem für die Forschung hilfreich sein. Einen bedeutenden Faktor für die Generierung von validen Testergebnissen stellt die Aufrechterhaltung der Aufmerksamkeitsspanne während der Testung dar. Diese sollte von den Untersuchern am Ende der Testung beurteilt und im Testprotokoll vermerkt werden. Im klinischen Rahmen sind derartige Faktoren von vornherein zu dokumentieren und im Sinne eines umfassenden „clinical reasoning“ zu bedenken [4].

### Zeitlich aufwendige Durchführung und kostspielige Anschaffung

Die Dauer der QST ist vom Testprotokoll abhängig. Da dieses jedoch nach Möglichkeiten das gesamte somatosensorische Profil erfassen sollte [3, 17], wird von der Verwendung eines zu wenig umfassenden Testprotokolls abgeraten. Im Gegensatz dazu gestalten sich lange Testdauern als wenig praktikabel und könnten die Konzentrationsspanne der Patienten übersteigen [17, 38, 50]. Die von der DFNS veröffentlichte Testbatterie stellt mit einer ungefähren Testdauer von 60 min ein kurzes und dennoch umfassendes Testprotokoll dar [50]. Dennoch existieren Bedenken, dass dieses Protokoll den gängigen zeitlichen Rahmen der klinischen Praxis übersteigen könnte [61]. Bislang konnte noch keine Studie bestätigen, dass die Durchführung der gesamten Testbatterie zwingend notwendig ist, um die erforderlichen Informationen zu erhalten. In diesem Zusammenhang wäre es interessant zu untersuchen, ob und unter welchen Umständen es möglich wäre, einzelne Tests in der Untersuchung zu überspringen, um wichtige Zeitressourcen einzusparen [7].

Für die Praxistauglichkeit zusätzlich erschwerend sind die hohen Anschaffungskosten für Geräte und Software [61]. Relativ kostengünstige Verfahren wie Testungen mittels Stimmgabel, Nadelstichen oder Monofilamenten können moderate bis schwere Sensibilitätsverluste relativ verlässlich erkennen [49], ihre Sensitivität reicht allerdings nicht für die (Früh‑)Erkennung von minimalsymptomatischen Neuropathien aus, welche sich häufig in Funktionsdefiziten der kleineren Fasern äußern. Einige der in der QST eingesetzten Geräte sind praktikabel, da sie als Handgeräte zur Verfügung stehen, weisen jedoch den Nachteil auf, nur spezifische Aspekte und nicht das vollständige Sensibilitätsprofil zu überprüfen. Geräte, die sämtliche somatosensorischen Funktionen austesten können, sind in der klinischen Praxis häufig kaum einsetzbar, da sie raumfordernd und kostspielig sind [49]. Momentan belaufen sich die derzeitigen Kosten einer QST-Testausstattung in der kostengünstigsten Variante auf rund 15.000 €, was den spärlichen Einsatz in den klinischen Praxen teilweise erklärt. Die umfassende Schulung der Untersucher, die im Sinne einer ganzheitlichen Standardisierung empfohlen wird, stellt einen weiteren Zusatzkostenpunkt dar, der zu berücksichtigen ist [7].

Vor Kurzem wurde das, laut Angaben der Hersteller, erste relativ kostengünstige Handgerät eingeführt, dass sowohl größere als auch kleinere Fasern hinsichtlich Funktionalität evaluieren kann. Dieses ist für etwa 500 USD zu erwerben und erzielte in einer Studie mit 130 Testpersonen gut reproduzierbare Ergebnisse, welche mit jenen von konventionellen QST-Geräten signifikant korrelierten. Mit einer hohen Sensitivität und einer moderaten bis hohen Spezifität könnte dieses Gerät eine kostengünstige und praktikable Alternative zu den häufig unökonomischen und sperrigen Apparaten der QST darstellen [49]. Erweist sich der Einsatz in der Praxis als zielführend, könnte dies die Entwicklung ähnlicher Geräte vorantreiben und so die ersten Schritte der QST in die klinische Routineanwendung ebnen. Um die diagnostische Treffsicherheit bewerten zu können, bedarf es allerdings noch mehrerer größer angelegter Studien. Außerdem sollte beachtet werden, dass die vorliegende Studie von den Herstellern selbst finanziert wurde.

### Limitierte Aussagekraft der QST

Zuletzt sollte hervorgehoben werden, dass der Aussagekraft der QST bestimmte Grenzen gesetzt sind. Ein gleichzeitiges Vorhandensein von Schmerzen und eines abnormalen sensorischen Profils kann die klinische Diagnose von neuropathischem Schmerz noch nicht mit Sicherheit bestätigen [58]. Anhand der QST kann ebenfalls nicht entschieden werden, ob detektierte Abnormitäten aufgrund von Neuropathien oder in Zusammenhang mit anderen nichtneuropathischen Geschehen auftreten [38]. Abnormale QST-Werte treten auch bei nichtneuropathischen Ereignissen wie arthritischen, myofaszialen oder fibromyalgischen Schmerzzuständen auf [4, 23]. Im Gegensatz zu anderen konventionellen Testmethoden ist die QST nicht in der Lage, die Stelle der neuralen Schädigung entlang der Neuraxis zu lokalisieren [3, 27, 57].

## Schlussfolgerungen

Zusammenfassend lässt sich sagen, dass die Ergebnisse und Inhalte der QST beträchtlichen Mehrwert für Physiotherapeuten und anderes medizinisches Personal in der Erkennung und Untersuchung von Neuropathien generieren können, was vor allem im Bereich der Small-fibre-Neuropathien umfassend durch Studien belegt wurde. Das Verfahren an sich weist bis dato noch einige Limitationen auf, welche den routinemäßigen Einsatz der QST behindern. Einige dieser Barrieren wie beispielsweise die unzureichende Standardisierung oder die Beeinflussung der Ergebnisse durch die Testperson können durch exakte Testausführung und Vorkehrungen bis zu einem gewissen Grad überwunden werden. Andere Schwierigkeiten wie die Verfügbarkeit von qualitativ hochwertigen Referenzwerten konnten durch die intensive Herausgeberarbeit der letzten Jahre bewältigt werden. Wieder andere – für die Klinik jedoch hochrelevante – Aspekte wie hohe Anschaffungskosten und der zeitliche Aufwand der Durchführung konnten bislang noch nicht zufriedenstellend gelöst werden. Weniger umfassende Testprotokolle [7] und die Entwicklung handlicher und kostengünstiger Testgeräte [49] stellen erste Lösungsansätze dar. Eine weitere, sehr praxisorientierte und kostengünstige Alternative ist es, die konventionelle BU um Testungen zur Wärmesensibilität und Schmerzschwellenbestimmung standardmäßig zu ergänzen. Auf diese Weise wäre die Evaluierung der kleineren, unmyelinisierten Fasern bis zu einem gewissen Grad gewährleistet, welche differenzialdiagnostisch eine wichtige Rolle spielen. Die Wärmediskriminierung mittels Münzen könnte dabei eine rasch durchführbare Möglichkeit darstellen. Die thermische Evaluierung mithilfe von Kleingeräten wie TipTherm® (Fa. TipTherm® GmbH, Wischhafen, Deutschland) [61, 64] oder NeuroQuick® (Fa. Schweers Informationstechnologie GmbH, Meerbusch, Deutschland) [69] stellt eine denkbare Variante dar. Eine alternative Schmerzschwellenbestimmung könnte beispielsweise über die Applizierung eines üblicherweise nicht schmerzhaften Fingerdrucks auf das Hautareal (statisch mechanisch) oder das Bestreichen der Teststelle mit einer Weichbürste (dynamisch mechanisch) gelingen. Für diesen Bereich existieren Kleingeräte, wie der Neuropen® (Fa. Owen Mumford GmbH, Großostheim-Ringheim, Deutschland), dessen Sensitivität in Studien bestätigt werden konnte [46]. Dass eine neurologische BU, sofern sie standardisiert abläuft und die Evaluierung von Qualitäten wie Wärme‑, Kälte-, und Vibrationsempfinden beinhaltet, durchaus geeigneter als eine QST im Hinblick auf die Evaluierung von neuropathischen Schmerzen sein kann, zeigt eine Arbeit von Timmermann et al. (2018; [61]).

Die angeführten Ergebnisse zeigen, dass vor allem der gut untersuchte Bereich der diabetischen Polyneuropathie von der Untersuchung einer QST im Sinne der Diagnostik aber auch Früherkennung profitiert. Wie diese Ergebnisse auf andere, bereits erwähnte klinische Erscheinungen umzulegen sind, muss noch eingehend untersucht werden. Baron et al. (2017) weisen in einer sehr umfassenden Darstellung auf die drei wesentlichen Komponenten hin:

- Sensorisches Defizit,
- Thermische Hyperalgesie,
- Mechanische Hyperalgesie.

Dabei ergeben verschiedene Formen der Neuropathie unterschiedliche Ausprägungen dieser Komponenten, die eine zusätzliche klinische Entscheidungsfindung unterstützen [5]. Nicht zu vernachlässigen ist im klinischen Rahmen der Einsatz von Fragebögen wie painDETECT, Douleur Neuropathique 4 (DN4) oder dem Leeds Assessment of Neuropathic Symptoms and Signs (LANSS), welcher die physiotherapeutische Untersuchung massiv unterstützen kann. Die standardmäßige Untersuchung der angeführten sensorischen Komponenten als Erweiterung der klassischen BU ist für den physiotherapeutischen Rahmen jedenfalls in höchstem Maße zu empfehlen. Neben der Möglichkeit eines präventiven Screenings profitiert letztendlich der betroffene Patient von einer zielgerichteten, multidisziplinären Behandlung, und einer Chronifizierung der Schmerzsymptomatik kann möglicherweise entgegengewirkt werden.
